# Family quality of life after brain injuries: a qualitative study on the perspectives of family members

**DOI:** 10.1007/s11136-025-04011-z

**Published:** 2025-07-04

**Authors:** José Luis Castillo, Alba Aza, María Fernández, Mari Storli Rasmussen, Nada Andelic, Miguel Ángel Verdugo

**Affiliations:** 1 Institute for Community Inclusion (INICO), University of Salamanca, Salamanca, Spain; 2 Department of Personality, Assessment, and Psychological Treatments, University of Salamanca, Salamanca, Spain; 3 Department of Physical Medicine and Rehabilitation, Oslo University Hospital, Oslo, Norway; 4 Faculty of Medicine, Research Centre for Habilitation and Rehabilitation Models and Services (CHARM), Institute of Health and Society, University of Oslo, Oslo, Norway; 5 Faculty of Health Sciences, Oslo Metropolitan University, Oslo, Norway

**Keywords:** Acquired brain injury, Family, Family-centered care, Family quality of life, Qualitative research

## Abstract

**Purpose:**

Exploring the perceptions of family members after an acquired brain injury (ABI) regarding their family quality of life (FQoL) is essential for tailoring interventions aimed at promoting recovery and improving family well-being. The aim of this study was to understand their perspectives on FQoL, identify its components, and differences among different family members of people with ABI according to their role within families.

**Methods:**

A qualitative study was conducted with ABI survivors and both primary (PCs) and non-primary family caregivers (NPCs) of adults with ABI. A total of 24 survivors, 22 PCs and 14 NPCs from different families were recruited through Spanish Brain Injury Federation. Eight role-specific focus groups on family well-being were conducted. Following verbatim transcription, a thematic analysis (Braun and Clarke, 2006) was performed to identify main domains of FQoL and their indicators. Comparative analyses examined differences among the three groups of participants.

**Results:**

Themes, subthemes and indicators of FQoL for ABI families were identified, encompassing five domains—*individual well-being*, *family relationships*, *family resources*, *community relationships* and *community resources—*. Also, differences between family members’ roles were reported.

**Conclusion:**

These findings highlight the importance of addressing FQoL by acknowledging the different family members’ experiences. From a family-centered approach, professionals should align to the goals and needs of different family members to provide appropriate support, aiming at enhancing the overall quality of life for families affected by ABI.

**Supplementary Information:**

The online version contains supplementary material available at 10.1007/s11136-025-04011-z.

## Plain English summary

When someone has a brain injury (e.g., strokes or traumatic injuries), families change their lives to support the person with brain injury. Some families may face challenges, so it is necessary to have interventions that address the needs and goals of all family members. In this study, we conducted meetings with people with brain injuries, family members who are the main caregiver and family members who are not the main caregiver. We wanted to know what family quality of life is for families of people with brain injuries. Aspects related to family well-being were summarized in 5 topics: (1) the well-being of each of its members, (2) the relationships between family members, (3) the family resources, (4) the relationships with people outside family, and (5) the resources for families in their community. This allows us to assess and create interventions aimed at helping families after brain injuries, adjusting to the well-being of each of the family members.

## Introduction

Acquired brain injury (ABI), including traumatic and non-traumatic brain injuries (e.g., strokes, hypoxias, brain tumors, or infections), is one of the leading causes of adult disability in Europe [1]. As a result, survivors may experience a broad range of long-lasting disabilities [2–4]. In Spain, the care provided to people with ABI is characterized by significant territorial fragmentation, leading to inequalities in access to rehabilitation and support services. Publicly funded rehabilitation is available during the acute phase within the healthcare system, while resources for the subacute and chronic phases are limited. In many cases, people with ABI and their families must rely on private services or support from family organizations, which can create financial and accessibility barriers [5].

In this scenario, families may try to adjust to the new situation to meet survivors’ needs. In this process, significant levels of anxiety, depression, health concerns, social isolation, or reduced levels of overall health-related quality of life have been described [6–11]. However, positive outcomes have also been identified, such as feeling appreciated, increased self-esteem and positive feelings [12, 13].

Most of the studies on the impact of ABI focus on primary caregivers, but its consequences likely affect the entire family. Families may face challenges such as poor family functioning [14, 15], relationship breakdowns [16], or financial hardship [17]. However, some studies have identified differences in how family members perceive family life according to their roles within families [18–20]. Thus, including family members beyond survivors and primary caregivers could provide a more comprehensive understanding of the family’s experiences [21, 22].

Given the complex needs and perspectives of family members after ABI, a family-centered approach has been proposed as the most effective approach in rehabilitation [23–25]. This model is defined as a coordinated system of community-based services for those with chronic conditions and disabilities, whose main goal is to achieve the maximum level of health and well-being of patients, their families, and communities [26]. However, there is an ongoing discussion about the objectives of this approach and how to integrate them into services. While some authors have focused on satisfaction with care, patients’ health, or caregiver’s health [25], Poston et al. [27] proposed Family Quality of Life (FQoL) as the main family-centered outcome [28], emphasizing the family as the core unit of care and the role of family-centered models in enhancing family well-being.

In the FQoL Theory, such a construct is conceptualized as “a dynamic sense of well-being of the family, collectively and subjectively defined, and informed by its members, in which individual and family-level needs interact” [28] (p. 262). It comprises individual and collective needs fulfilment, shared-time enjoyment, and opportunities to achieve their goals [27, 29]. This concept is based on individual quality of life theory, family empowerment and socioecological models of disability [30, 31].

Like on Individual Quality of Life, FQoL is considered a multidimensional construct, defined by interrelated domains which are operationalized through indicators such as perceptions, behaviors and life conditions at micro-, meso-, and macrosystems level, which serve as both factors and outcomes of individuals and families wellbeing ([32, 28]). Several groups have attempted to develop assessment tools which help to identify indicators and measure FQoL. The International Quality of Life Project developed and validated the Family Quality of Life Survey (FQoLS-2006) [33], which assess FQoL across nine domains (i.e., *Health of the family*, *Financial well-being*, *Family relationships*, *Support from other people*, *Support from disability-related services*, *Influence of values, Careers*, *Leisure and recreation*, and *Community interaction*). This survey was developed with international experts and validated through pilot testing with families of individuals with intellectual and developmental disabilities (IDD) across several countries [34]. Similarly, the Beach Center Family Quality of Life Scale [35] was created through an extensive work with families of children with IDD, as well as the development and validation of the scale in the USA [27, 36]. Despite their differences, a key feature is the emphasis on the perspective of family members to define which indicators best reflect family well-being, thus recognizing and understanding their experiences.

The FQoL approach to study family members’ desired outcomes may be of interest for comprehensively assessing family well-being and planning family-centered interventions, services and policies [30, 37]. However, few studies have been identified on the FQoL following ABI. Although these studies have tried to understand FQoL from the perspective of primary caregivers [38, 39], they failed to include the whole family system. Therefore, research on the perspectives of the different family members on their FQoL is needed. A study with ABI survivors, primary caregivers (PCs) and non-primary caregivers (NPCs) was conducted to better understand (a) their perspective on FQoL, (b) which indicators represent the FQoL and, (c) whether there are differences between their reflections according to their role. To achieve this aim, qualitative methodology was chosen since it allows to build situated and deep knowledge of social life from the perspective of stakeholders, and it has been proposed as an essential approach to understand people’s experiences of well-being [40].

## Methods

### Recruitment

Participants were a convenience sample of survivors, PCs and NPCs. Seven local family organizations of individuals with ABI across the country were contacted to collaborate in the study. Participation was offered to survivors and relatives of people with ABI by staff members. A maximum variation strategy was used for recruiting participants as we wanted to capture the widest range of family situations possible.

Participants were adult survivors and family members of people with ABI receiving services from the collaborating organizations. None of the participants belonged to the same families. Supplemental Data File 1 described the inclusion/exclusion criteria for participation.

### Sample characteristics

A total of 60 participants took part in the study. Three focus groups with 24 survivors, three with 22 PCs and two with 14 NPCs were carried out. Tables 1 and 2 display participants’ characteristics.

**Table 1:** Sociodemographic characteristics of participants

	Survivors	Primary caregivers	Non-primary caregivers
	N (%)	N (%)	N (%)
No. of Focus groups	3	3	2
Participants by focus groups			
Mean	8.00	7.33	7.00
SD	0	2.31	1.41
Range	8	6–10	6–8
Age			
Mean	50.08	54.50	42.50
SD	12.20	11.62	16.22
Range	23–70	25–72	19–71
Gender (n = 60)			
Female	11 (45.80)	21 (95.50)	8 (57.10)
Male	13 (54.20)	1 (4.50)	6 (42.90)
Educational level			
No school certificate	1 (4.20)		
Elementary	6 (25.00)	3 (13.60)	3 (21.40)
High school	12 (50.00)	12 (54.50)	5 (35.70)
University	5 (20.80)	7 (31.80)	6 (42.90)
Pre-injury/Current employment status			
Working	19 (79.20)/0	12 (54.50)/4 (18.20)	10 (71.43)/9 (64.30)
Studying	1 (4.20)/1 (4.20)	1 (4.50)/0	1 (7.10)/1 (7.10)
Unemployed	2 (8.30)/2 (8.30)	4 (18.20)/10 (45.50)	0/1 (7.10)
Unable to work	0/14 (58.30)	1 (4.50)/1 (4.50)	1 (7.10)/1 (7.10)
Retired	0/7 (29.17)	3 (13.60)/5 (22.70)	2 (14.30)/2 (14.30)
Other	1 (4.20)/0	1 (4.50)/2 (9.10)	0/0
Income (EUR per month)			
Less than 500	2 (8.40)	7 (31.80)	1 (7.10)
500–1,000	5 (20.80)	3 (13.60)	2 (14.30)
1,000–1,500	8 (33.30)	5 (22.70)	5 (35.70)
1,500–2,000	3 (12.50)	5 (22.70)	4 (28.60)
2,000–2,500	2 (8.30)	2 (9.10)	2 (14.30)
More than 2,500	2 (8.30)		
Preinjury/Current civil status			
Married/with partner	16 (66.70)/14 (58.30)	18 (81.80)/19 (86.40)	7 (50)/5 (35.7)
Single	4 (16.70)/5 (20.80)	3 (13.60)/2 (9.10)	4 (28.60)/4 (28.60)
Separated/divorced	3 (12.50)/5 (20.80)	1 (4.50)/1 (4.50)	0/2 (14.30)
Widow/widower	1 (4.20)/0		3 (21.40)/3 (21.40)
Relationship			
Spouse/partner		15 (68.20)	0
Parent		2 (9.10)	5 (35.70)
Children		2 (9.10)	0
Sibling		3 (13.6)	3 (21.40)
Other*		0	6 (42.86)
Number of family members			
Mean	4.04	3.77	5.23
SD	1.40	1.48	2.28
Range	2–7	2–7	3–11
Living condition			
Living with survivor		20 (90.90)	5 (35.71)
Not living with survivor		2 (9.10)	9 (64.29)
Survivor’s living arrangement			
Family home	19 (79.2)	22 (100.00)	9 (64.30)
Independent living	3 (12.5)		
Residence			5 (35.70)

^*^Other included type of relationships such as cousins, sibling- and children-in-law

**Table 2:** ABI’s characteristics

	Survivors	Primary caregivers	Non-primary caregivers
	N (%)	N (%)	N (%)
Time since onset (years)			
Mean	8.69	11.20	15.35
SD	5.42	11.75	17.56
Range	2–20	1–27	2–61
Aetiology			
Cerebrovascular accident	13 (54.20)	9 (40.90)	6 (42.90)
Traumatic brain injury	8 (33.30)	6 (27.30)	5 (35.70)
Cerebral anoxia	1 (4.20)	4 (18.20)	1 (7.10)
Cerebral tumours	1 (4.20)	3 (13.60)	1 (7.10)
Infectious disease	1 (4.20)	0	1 (7.10)
Current health conditions			
Physical disability	16 (66.67)	20 (90.10)	11 (78.57)
Cognitive deficit	17 (70.80)	21 (95.50)	11 (78.57)
Sensory disability	10 (41.67)	9 (40.90)	4 (28.57)
Language and communication	6 (25.00)	12 (54.50)	6 (42.85)
Mental health problems/emotional disorder	13 (54.20)	12 (54.50)	4 (28.57)
Behavioural problems	8 (33.30)	17 (77.30)	3 (21.43)
Associated chronic pain	4 (16.67)	8 (36.40)	1 (7.14)
Epilepsy	5 (20.83)	10 (45.50)	1 (7.14)
Others	1 (4.17)	1 (4.50)	0

Note: ABI’s characteristics are related to both survivor participants, and survivors who did not take part in the study but their family members (i.e., PCs or NPCs)

### Data collection

Following verbal and written consent, participants were asked to complete a self-reported form with sociodemographic information. To collect participants’ experiences, focus group technique was chosen as it may provide deeper information within the interaction among participants. Based on previous studies about families post-ABI and FQoL assessment tools, a semi-structed guide was designed to obtain information about participants’ perspectives on FQoL, contributing factors to either good or poor family well-being and how these aspects had changed since onset (see Supplemental Data File 2).

Face-to-face focus groups were carried out in local organizations’ facilities ensuring privacy and comfort. They included six to ten participants and lasted approximately one hour and a half. Focus groups were role-specific (i.e., survivors, PCs, and NPCs) to facilitate disclosing and compare family members’ perspectives according to their roles. Special attention was given to building a good rapport during focus groups since no previous relationship existed between researchers and participants. In this sense, all participants’ viewpoints were considered valuable and valid. Facilitation strategies, such as breaks, reformulations or eliciting peer support, were used when needed. Focus groups were audiotaped and transcribed verbatim.

### Data analysis

Thematic analysis was used for data analysis [41, 42]. The analysis was influenced by family and individual QoL theories, the systemic approach and the family empowerment model [28, 30]. Codes and themes were inductively generated based on the descriptions provided by participants. Reflections about personal and professional experiences with disability, ABI and QoL were registered by researchers through field notes and memos to understand analysis’ influences and its construction. A description of analysis phases is displayed in Table 3.

**Table 3:** Phases of analysis

Phase	Description of process
0. Data collection and transcription	Eight role-specific pre-established focus groups in the native language of participants (i.e., Spanish) were carried out in local organizations facilities by JLC and AA
1. Familiarizing with data	Focus groups were audiotaped and transcribed verbatim by JLC and a master’s degree student. Field notes with reflections while data collection and transcriptions were also recorded. Transcriptions were read and re-read in order to immerse in the data by JLC and AA
2. Generating initial codes	Through MAXQDA, codes were inductively generated according to patterns identified in transcriptions, considering the surrounding data by JLC and AA, first individually and by discussion
3. Searching for themes	Codes were reviewed and compared across transcriptions in order to create preliminary overarching themes by JLC
4. Reviewing themes	Themes were checked back in relation to extracts coded as well as the whole data set by JLC and AA. Disagreements were solved by discussion with MAV
5. Defining and naming themes	Names and descriptions of themes were generated to explain the meaning of results by JLC. Information has been reviewed while preparing this manuscript
6. Comparisons among role-specific focus groups	Comparative analysis among the three types of groups (i.e., survivors, primary caregivers and non-primary caregivers) were conducted by JLC and AA to examine whether participants’ perspectives on specific QoL indicators differed according to their roles. This involved identifying similarities and differences in the descriptions of the indicators associated to each theme and sub-theme provided by participants according to their roles
7. Producing the report	A preliminary report of results has been produced. Results were discussed with stakeholders (i.e., other researchers on family, ABI rehabilitation and QoL, as well as leaders of ABI organizations) as member reflections Findings were translated to English and checked-back for accuracy by JLC and AA, as they better understood the setting in which data collection and analysis were carried out. The remaining authors reviewed the meaning of findings and quotes in English The current study was produced to report results, reviewing and checking back the analysis

Adapted from Braun & Clarke [42]

To ensure rigour, credibility, transferability, and dependability, several procedures have been considered [43]. For the former, data were triangulated in three ways. Multiple perspectives of family members were considered (i.e., survivors, PC and NPC), as well as multiple locations around the country (i.e., local organizations of the Spanish Federation of Brain Injury) and multiple researchers have participated in data collection and analysis. Also, results were discussed with researchers on family, ABI rehabilitation and quality of life, as well as with leaders of ABI organizations. While researchers help to establish the linkage between the current study and FQoL and Care theories, ABI organizations’ leaders provided important insights regarding codes relationships and theme clarification. In the case of transferability, maximum variation strategy was used to ensure that a wide range of characteristics was represented. For dependability, the research team have broadly discussed the meaning of the findings in data collection and analysis, as well as the analysis has been carried out by reflecting on the experiences and knowledge of researchers.

## Results

**Fig. 1:**
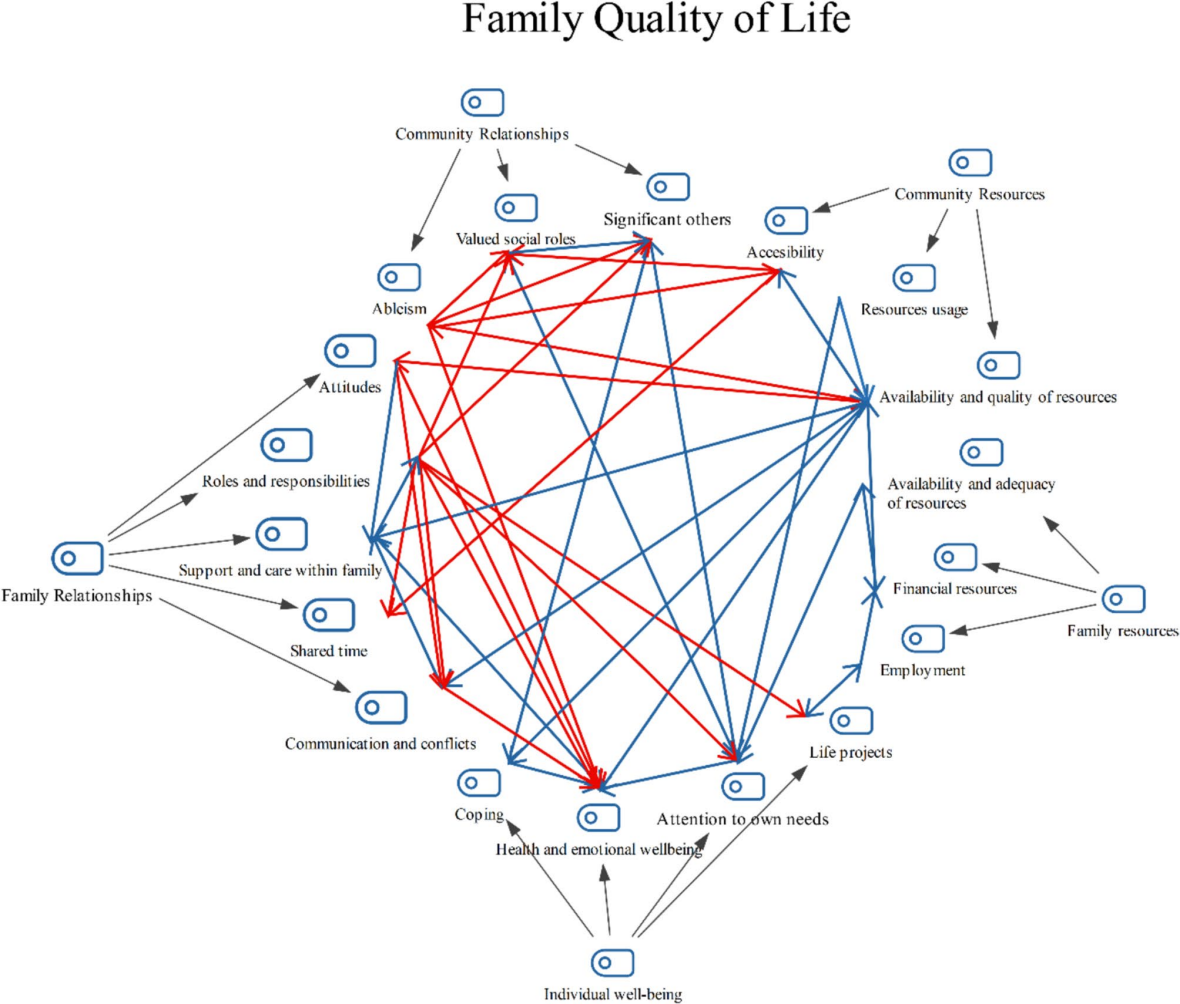
Coding structure with co-ocurrence of subthemes

A two-level coding structure has been generated as a result of the analysis. This structure describes a set of FQoL domains and indicators for this population. Table 4 displays indicators and quotes illustrating themes and subthemes. Also, differences in participants’ accounts according to their role (i.e., survivors, PCs, and NPCs), and subthemes relationships have been identified (see Fig. 1).

**Table 4:** Family Quality of Life themes, subthemes and indicators with quotes

Subthemes	Indicators	Quotes
**Theme 1. Individual wellbeing**		
*Health and emotional wellbeing*	Enjoy good health	*“If there are no illnesses, everything is fine […]. We talk about everything regarding health”* (NPC2406)
	Need for professional support	*“But, above all, psychological support also for them, because (for) them, the family that is with you, that lives with you or that takes care of you, is a huge blow and managing that, managing that change of life for them is also…, it's necessary”* (S1602)
	Accept oneself	*“When you said, ‘I have distanced myself from my daughter because I didn’t want to be a burden for her’, why do you think you are a burden? You are different from what you were, but you are not a burden”* (S1602)
	Have positive/ negative feelings	*“We have a lot of breakdowns, we are mad at the world [for what has happened]”* (PC1202)
*Coping*	Use proper coping strategies to face difficulties	*“It’s trying to find the good side of it, such as being able to take care of my mum, when the time I was there and, well, trying to adapt to what life offers”* (NPC2404)
*Attention to own needs*	Meet own needs (e.g., basic needs, health needs…)	*“They need to allocate responsibilities and take care of them themselves too because otherwise it’s exhausting”* (NPC2802)
	Have time for themselves	*“They told us about [name of the organization] and that’s where he went. My salvation because it’s the need to respite a bit, to have your own life, isn’t it?, what the caregiver needs and with that I was able to have a bit of time [for myself]” (PC1201*
	Be involved in pleasant activities	*“The only thing that takes my mind off is sports, so I joined [the gym] and I enjoy it a lot”* (PC2406)
*Life projects*	Be satisfied with own life	*“[Talking about what is important for their lives as individuals] You see them enjoy it, laugh. Live, live [laugh]”* (PC2401)
	Have personal goals and opportunities to achieve them	*“For me, [date of onset], when he had the stroke, his life changed, but I, my life has changed as well, my dreams vanished. For four years, everything was him and the kids, I didn’t matter”* (PC2404)
**Theme 2. Family relationships**		
*Support and care within family*	Receive support when needed	*“Although she lives far away, my sister is always there, with all the legal stuff, she is always there”* (S0108)
	Know how to support	*“[Talking about extended family] I think they would also feel part of this, of this situation, right? Because I know that they…, for example, they want to help, but they don’t know how to”* (PC1702)
	Reciprocal support	*“So, it’s this sort of thing, which is frustrating for family members, we all support each other, but we cannot count on her anymore, although we always had” (NPC2805)*
*Roles and responsibilities*	Be satisfied with their roles	*“At home, we have done everything together, I’ve never had troubles with ironing or cooking, and now I am the housekeeper. And I love it, phew, I love it!” (S1601)*
	Be able to attend responsibilities	*“[Talking about caring and other responsibilities] In my experience, there’s a moment when you say, ‘I can’t anymore’, we are no longer able to keep up with everything”* (NPC2801)
	Fair distribution of responsibilities	*“Regarding roles, every, everything changes. The entire responsibility falls on me. And now, you are the one who is in charge of everything”* (PC1704)
*Shared time*	Be involved in family activities	*“My wife and my siblings are planning a family meeting for the summer, and I always have a go at anything!”* (S2301)
	Share interests	*“He [survivor] participates in cycling races with the kids. They go all together”* (PC2404)
	Enjoy time together	*“The truth is that the time we spent with my mother-in-law, reached a point where it was no longer a time of quality, wasn't it? It's many hours, it's caring, that's it, everything” (NPC2806)*
*Communication and conflicts*	Know how to interact with each other	*“It took a lot, for example, in the case of my daughter. But, bit by bit, they have learnt to…, to interact in another way”* (CP1704)
	Frequent conflicts	*“I would say that, of course health is important, but it’s also important for families to not have conflicts, because there is always a conflict”* (S2301)
	Be involved in family decision-making processes	*“Decisions have to be made. We [parents of the survivor] do involve her sister so that she’s aware, but we make the decisions ourselves”* (PC1205)
	Share things (e.g., information, opinions, ideas) with each other	*“My parents previously had conversations, I don’t know, like ‘[Participant’s name] had an exam’. I found it very important”* (NPC2804)
	Express affection	*“Since this happened to him, he has become…, he is dependent, but very close to the family, which he wasn't almost before, he wasn't, and now he is very affectionate and all”* (PC2403)
	Feel understood	*“(She) is… the person who… I argue the most, and… I think that… they don’t, they don’t understand the illness that we have. They don’t understand it”* (S0107)
*Attitudes towards other family members*	Feel valued by others	*“I have always felt loved and supported by my siblings and my mother”* (NPC2801)
	Positive/negative attitudes towards other family members	*“¿My father? [I am] childish for my father. With his friends, ‘Oh! [Name of participant], oh! Come on…’ [Imitating the tone of infantilization with which she received such comment]”* (S0101)
**Theme 3. Family resources**		
*Availability and adequacy of resources*	Enough resources to meet family members’ needs	*“When he was told that he would never again be able to ride a bike because of the hemiplegia, it was a shock. He has bought an adapted bike”* (PC2404)
	Barriers at home	*“I was living on the second floor without an elevator. It was a big deal”* (S1601)
	Adapting family places to members’ needs	*“Adapted shower tray. I have a seat in the shower, and I shower there. It’s adapted, we spent the money on that [adapting the bathroom]”* (S0103)
*Financial resources*	Balance between income and expenses	*“If you have small children, it’s impossible with the money [of his disability benefit] I’m paid. It’s impossible”* (S1604)
	Be able to afford rehabilitation services	*“[Talking about the services provided by the ABI organization] At first, we could not afford it because it was private and costly for us”* (PC1205)
	Adjust expenses	*“[Talking about finances] You need to adapt to what you are able to [afford]. If you have less [money], you should make an effort and spent less”* (S2308)
*Employment*	Quit jobs	*“I had to quit my job, but I advise to everyone not to quit, to try [keep working]”* (PC1702)
	Balance between family life and work	*“I requested a reduction of working hours, well, from 40 to 35, not a big deal… But, I needed a morning schedule in order to care for my children”* (PC1703)
**Theme 4. Community relationships**		
*Valued social roles*	Activities that fit family members’ needs and preferences	*“They [his friends] see your disability as a barrier for, for example, going out ‘till 6AM or… You can’t just keep up with alcohol”* (S0105)
	Be involved in community activities (e.g., social events or leisure)	*“At least in my case, you stop doing certain activities and you only do that things you know will work with him”* (NPC2802)
	Enjoy community activities	*“I’m thinking in big events. People is having fun, and you just stay alert in case something happens”* (NPC2801)
	Perform socially valued activities	*“He [survivor] participates in cycling races […] and people in the club cheer him up and all”* (PC2404)
*Significant others*	Have opportunities to engage in activities with people outside family	*“You see, money is necessary for hiring someone who can take care of him [survivor] at weekends so that I can go on a trip with friends”* (PC1201)
	Have meaningful relationships with people outside family	*“[Talking about friends] That they come to visit you, that you can talk with someone else or just spent an evening. It’s so important. I was lucky that I have friends who were more than family”* (S2301)
	Feel isolated	*“[Talking about barriers in communication with people outside family] He is aware, so many times he asks me ‘Am I that bad to be that lonely?’* (CP1702)
	Be satisfied with social relationships	*“But I think I am lucky for having almost the same friends [as she had pre-injury] and we have grown up together”* (NPC2405)
	Receive different types of support from people outside family when needed	*“[Talking about neighbors] We are a little family that we care each other a lot […]. We don’t have any complaint, we feel very loved by everyone, and they have always been available if we needed help”* (NPC2804)
	Feel understood	*“In my case, my friends have always understood [the situation], they have always showed care for me”* (PC1204)
*Ableism*	Be respected	*“In my case, the response has been so positive, people have always been so respectful”* (PC1201)
	Experience discrimination	*“When my daughter came from school, she was always crying, ‘What happened, sweetie?’, and she said, ‘In the school, I was told that you are dying’, I replied ‘No, don’t worry’”* (S1605)
**Theme 5. Community resources**		
*Availability and quality of resources*	Available high-quality resources (i.e., general and specialized social and healthcare resources)	*“In the neighborhood, I don’t see any problem. However, the community resources should do better […]. When you go to social care services, healthcare, these resources should have information, know, understand or do things better, but they do it worst than the broader community”* (PC1206)
	Receive services when needed	*“If there’s the case that my health and life or my loved ones is at risk, it’s fundamental. Talking about social and health care services, that they fulfill all [family needs]”* (S2301)
	Tailored services to family needs	*“Many times, in a gym, a pool, or leisure services, there is a person for everyone, and there’s no support staff”* (NPC2405)
*Accessibility*	Barriers to access and enjoy services	*“Recently, I went to the [name of the city] courthouse and fortunately it’s being changed because I said, ‘In that elevator, ¿how does a wheelchair go in?’ Things of public places’ design that should take into account everyone and it does not”* (NPC2804)
*Resources usage*	Have information about community services	*“I’m afraid that I can no longer take care of my children because of the stroke […]. We don’t have information about how to manage certain things, for example, where to look for help”* (S0108)
	Know how to choose or contact services that fit family needs	*“There may be resources, but just like that one there are 20 more. There are families that have money, but they don’t spend that money in what they should do […]. You can take him [survivor] to a place where he is not receiving what he needs”* (PC1710)

^*^Quotes are displayed along with identifiers – S for survivors, PC for primary caregivers and NPC for non-primary caregivers

### Individual well-being

This domain captures the well-being of each family member, including subthemes such as *health and emotional well-being*, *coping*, *attention to own needs* or *life projects*.

*Health and emotional well-being* encompasses positive and negative outcomes related to physical and psychological well-being, as well as the need for professional support. Apart from the health status of survivors, participants especially described mental health issues (e.g., anxiety or depression) for the rest of the family. This was linked to descriptions of *coping* post-ABI, specifically participants referred to strategies to deal with their new lives.

On the other hand, participants spoke about *meeting their own needs* such as basic needs, healthcare needs or other type of needs (e.g., leisure). In this sense, the most frequently described aspect was how family members lacked time for caring for themselves because of caregiving responsibilities, specifically among PCs. This didn’t describe only family members’ needs, but meaningful *life projects* as well.

### Family relationships

Participants described aspects of the interaction among family members, such as *support and care within family*, *roles and responsibilities*, *shared time*, *communication and conflicts*, or *attitudes towards other family members*.

The first subtheme was one of the most cited issues to FQoL. While PC and NPC described the disparate involvement of other family members in caring for survivors and its impact on their emotional well-being (e.g., feelings of disappointment, anger, and resentment), survivors expressed gratitude for the support received. Additionally, some participants across all focus groups acknowledged the importance of reciprocal support between family members and how it had changed for some of them.

Participants frequently linked support to *roles and responsibilities*. Family members described the process by which family roles were redistributed. As a part of FQoL, participants described satisfaction with these roles. For many PC and NPC, these were experienced as a responsibility and some of them referred to the strain posed by juggling among them. By contrast, other family members highlighted their satisfaction with their current situation.

Third, participants spoke about *shared time*. Family activities were expressed to be constrained by different factors. PC pointed to caregiving and other responsibilities as causes for the limited time to engage in them. Along with NPCs, they also discussed how caregiving and vigilance in case of potential crisis hindered their enjoyment. Additionally, all participants linked this to the presence of barriers in the community.

Another subtheme related to FQoL was *communication and conflicts*, which describes how families make decisions, share information (e.g., day-to-day issues, emotions or affection), and sources of conflicts and how to cope with them. When it came to decision-making, participants differed in how other family members, aside from PC, participated in such processes. However, PC and NPCs agreed that while survivors’ needs were considered, survivors themselves were not actively involved.

Regarding the last subtheme, *attitudes towards other family members,* participants referred to receiving enough attention and being valued by the rest of the family. In all groups, different attitudes towards survivors were discussed, varying from rejection and avoidance, through patronizing, to acceptance of the new life situation and expressions of affection and appreciation. By contrast, NPCs spoke about how the lack of appreciation for themselves was associated with feelings of being left out or absence of value.

### Family resources

This domain describes resources that families use to fulfill their members’ needs. Participants described different types of resources in three subthemes: *availability and adequacy of resources*, *financial resources,* and *employment*.

In the case of *availability and adequacy of resources*, participants referred to a wide range of goods necessary to fulfill family needs as well as its adequacy. It included those related to basic needs (i.e., food or housing) and other types of needs (i.e., transportation or leisure material), as well as assistive products. Survivors and NPC noted that many preinjury goods didn’t fit with the new family situation, thus modifications were needed.

On the other hand, all groups discussed the importance of *financial resources* to access these resources and rehabilitation services. Participants described financial hardships as a result of rehabilitation expenses and reduced sources of income, which was linked to the *employment* of survivors and PC, who had to resign from their jobs, request reductions in working hours or sick/family leaves.

### Community relationships

Participants acknowledged the importance of the relationships that family members established within their communities for FQoL. Most of them described topics related to *valued social roles*, *significant others,* and *ableism*.

Regarding the first, participants raised the need to be involved in socially valued activities beyond family (i.e., activities that contributed to others, activities that were important in their communities). Some participants noted that certain activities didn't meet survivors’ needs, preventing the family from participating due to accessibility issues as well as social interactions (e.g., physically demanding activities, alcohol consumption, or noise). In this sense, survivors referred to how important it was to take part in leisure with friends, while PC and NPC described the lack of time to do so, the enjoyment of activities, and the feeling of guilt.

In the case of *significant others*, participants described relationships with people outside the family system (e.g., friends, colleagues, neighbors, acquaintances and other people). All groups discussed the restrictions to their social networks post-injury and not feeling satisfied, especially for survivors and PC. These changes were linked to reductions of support, although it was highly praised when available. However, some participants reported establishing new relationships with people from ABI organizations, as they felt more comfortable and understood.

Regarding *ableism*, participants cited aggressions experienced within the community (e.g., talking to family members instead of directly to survivors), which not only had an impact on survivors, but the rest of the family.

### Community resources

This domain covers the full range of resources within communities for meeting family needs. It includes themes such as *availability and quality of resources*, *accessibility*, and *resources usage*.

For the first, all groups raised the need for communities to have available and high-quality resources that meet family needs. Specialized social and healthcare resources’ quality (e.g., rehabilitation services, financial aid and benefits or in-home support services) were characterized by three main aspects: (1) the specific knowledge and training of their professionals, (2) empathy and respect for survivors while working with them, and (3) timely-accurate addressing of families’ needs. In addition, some PCs spoke about the impact on their relationships with survivors when these resources lacked.

In contrast, only NPCs and one group of PCs expressed the need for adequate communities’ general resources to all family members (e.g., primary care, justice or cultural, recreational and sporting services). These should be tailored to families interests and characteristics and promote inclusion and well-being of all family members. However, this was thought to be restricted by *accessibility* barriers for survivors and their families. While PCs expressed that bureaucracy hindered their ability to access specialized services, survivors and NPCs focused on physical and cognitive barriers in general resources.

Also, the *usage* of these very services was described. Participants spoke about the knowledge and information that families have about these services, as well as how they choose and use them to meet their needs. Many participants described having felt helpless after hospital discharge.

In this figure, a MAXQDA hierarchical coding structure is displayed. FQoL is described by five themes or domains and their subthemes. Relationships between subthemes have been established according to the co-occurrence of subthemes’ coded extracts. The red arrows indicate negative relationships, while the blue arrows indicate positive ones, both following the direction of the arrows as derived from the coded segments.

## Discussion

This study attempted to understand FQoL from the perspective of individuals who belonged to families of people with ABI according to their role and to identify indicators suitable for this population. Through a TA of focus groups with family members of adults with ABI, an operational multidimensional model of FQoL has been proposed. This model accounts for five domains of FQoL -namely, individual well-being, family relationships, family resources, community relationships and community resources- with indicators across individual, family and community levels. These domains and indicators are interconnected defining family factors and desired outcomes. Moreover, differences in participants’ accounts about FQoL according to the role they hold within the family (i.e., survivors, PCs, and NPCs) are reported.

One of the core domains identified in the model was *individual well-being,* where participants emphasized factors such as physical and psychological well-being. Psychological concerns were the most frequently mentioned indicators, aligned with existing literature on desired family outcomes post-ABI [12, 44, 45]. Although studies on the physical consequences of caregiving are also abundant [46], participants spoke about physical well-being mostly related to survivors. This may be because changes in physical well-being post-ABI may be more apparent in survivors. Yet, it is a major concern for all family members [47].

On the other hand, when asked about family well-being, the most frequently described topics were related to *family relationships*, addressing the exchanges and interactions among family members [34, 36, 48, 49]. Some aspects, such as *communication and conflicts* or *shared time*, have been highlighted. However, support within the family, both for the person with ABI and for other family members, was the most described subtheme. These findings differ from those other studies with children with disabilities which incorporate support as a different topic within *family roles* domains, which entails the ability of families to provide support for people with disabilities [50]. This cannot be fully applicable to adults with disabilities as it may stress the responsibility of caring within the family, which is frequently expected to be taken on female family members. But it also suggests that support is only directed towards people with ABI, which may be inappropriate and patronizing. In this sense, a discussion about mutual support emerged among NPCs and survivors’ groups. This has previously been acknowledged when people with disabilities are included in studies about FQoL [51].

Despite its importance, varying degrees of support and involvement were mentioned as well as its impact on individual well-being, especially when lacking. This has also been reported in previous studies with ABI survivors [15], and is one of the aspects where family members’ perceptions most frequently disagree [19]. In this sense, distribution of care labor within families is linked to better mental and physical health outcomes [52, 53]. Thus, promoting involvement other than PCs and mutual support in the context of family interventions may enhance relationships and, consequently, the emotional well-being of family members.

Another significant aspect of FQoL was *family resources*, which has been discussed either as material or financial well-being [50]. Like in our study, finances seem to be the main source to fulfill family needs. This is a particularly important issue for this population due to the decreased income post-ABI and the rising expenses related to rehabilitation needs, which may hinder the psychological adaptation of family members as well [17]. Nevertheless, participants also reflected on the usage of these resources when referring to the process by which family resources were adapted to the new family situation. In fact, this aspect has been described by Engel et al. [54], where survivors access, plan, manage and make choices related to their resources. This paper studied the contextual factors that influence financial well-being post injury and how the access to resources was constrained by societal factors, like those described in the *community resources* domain of this study.

In the case of *community relationships,* groups particularly discussed interpersonal relationships and support of people beyond family. In this regard, the loss of meaningful bonds was referred to be connected to a reduction of support sources, often resulting in feelings of abandonment and loneliness, especially for survivors and PCs. While this has been a well described topic in the previous ABI literature [55, 56], some participants described situations in which pre-injury relationships remained or new ones were stablished with other survivors and ABI families, which is in line with the findings of Salas et al. [57].

On the other hand, this domain did not only refer to these relationships but also participation in the community in a broader sense. These aspects have previously been highlighted under *Community interaction* and *Support from other people* domains [58], which represents how family system interacts with friends, neighbors, and other people within their communities. In this study, participants referred to the importance of activities and performing valued roles outside the family, although withdrawal was frequent due to barriers to participation (e.g., time constraints, physical and cognitive barriers). This suggests that other members beyond survivors may also be indirectly exposed to these barriers. Despite this aspect having previously been described as a desire outcome for family members [59], it remains as a frequently neglected area within family interventions [60]. Thus, incorporating specific strategies to overcome social barriers and enhance connections with the community not only for survivors but for all family members may strengthen the impact of family interventions.

For the last domain, one of the major concerns across all groups about FQoL was the availability of specialized social and healthcare services that adequately meet family members’ needs as in the study of Climent et al. [39]. Also, general community resources were referred by survivors and NPCs. Community resources and, especially, disability-related services have been an important topic across FQoL studies [61]. Additionally, some family members acknowledge the importance for families of using such services as they have faced some challenges navigating social and healthcare systems [62]. Similarly, in the current study, participants reflected upon the need of information about services that helped them to navigate the social and healthcare system, which may be an important aspect not only for survivors’ rehabilitation, but also for all family members’ needs.

## Limitations

This study has identified desired outcomes regarding FQoL by analyzing the perspectives of ABI adult family members according to their role. In this sense, differences have been noted between the current proposal and other models (e.g., [36, 48]). However, quality of life indicators and its importance are thought to be culturally and contextually sensitive [63, 64]. Thus, it is important to emphasize that some indicators may not be applicable to other populations or countries, since the way families relate, their priorities and social and healthcare systems may differ across countries.

Moreover, the findings of this study may be influenced by the sample included. On one hand, some groups (i.e., PC) were mainly composed of female family members. Because differences in the experience of female and male caregivers have been identified [65, 66], some of the described indicators, their importance, or perceived changes are likely to vary for male PCs, especially for roles and responsibilities. This may be similar to the case of the NPCs who participated since they were family members that somehow were involved in caring and supporting survivors, which may differ from family members who don’t take part of family caring tasks.

## Conclusions

While we acknowledge these limitations and the differences with other proposals, and therefore the need to explore this structure further, this study can guide FQoL assessment and target interventions aimed at improving family well-being at individual family, health care and family services, and policy levels. Overall, the study findings suggest that FQoL is a multidimensional construct and comprises interconnected indicators within five domains (i.e., *individual well-being*, *family relationships*, *family resources*, *community relationships* and *community resources*). Further, FQoL indicators differ according to the role of the family member. While ABI survivors frequently described individual-level indicators, PC and NPCs referred to their relationships with survivors and other family members. In this sense, NPCs were more likely to reflect upon family conditions as a system. These indicators may also vary across contexts, cultures, and family members, stressing the necessity of focusing on the experiences of all relatives. This requires assessment tools that not only gather the perspective of the different members of the family in relation to their well-being, but also integrate them in such a way that the needs of the whole family can be addressed. This can help to understand family members’ needs and how they are linked to develop rehabilitation and family interventions that promote the family well-being by adjusting to these needs.

## Supplementary Information

Below is the link to the electronic supplementary material.

Supplementary file 1 (DOCX 244 KB)

Supplementary file 2 (DOCX 244 KB)

Supplementary file 3 (DOCX 262 KB)

## Data Availability

Focus groups transcriptions are not available.
