# Supplementary material for: Family quality of life after brain injuries: a qualitative study on the perspectives of family members
Source: Qual Life Res. 2025 Jul 4;34(9):2701–17. doi: 10.1007/s11136-025-04011-z (PMC12432037; doi:10.1007/s11136-025-04011-z)
Supplement: Supplementary file 1 — (DOCX 244 KB) [file 11136_2025_4011_MOESM1_ESM.docx]

**Family quality of life after brain injuries: A qualitative study on the perspectives of family members**

*[Author information – Anonymized]*

# Supplemental Data File 1. Inclusion/exclusion criteria for participation

**Table 1.** Participant’s inclusion/exclusion criteria by role.

|  | **Survivors** | **Primary caregivers** | **Non-primary caregivers** |
| --- | --- | --- | --- |
| **Definition** | People that have had a brain injury (i.e., cerebrovascular accident, traumatic brain injury, brain tumour, anoxia or brain infection) and have remaining health conditions | Relatives of survivors who provided most care within families | Relatives of survivors did not hold the PC role but could be somewhat involved in caring tasks |
| **Inclusion criteria** | Had had a brain injury at least one year ago | Their relative with ABI had had a brain injury at least one year ago | Their relative with ABI had had a brain injury at least one year ago |
|  | At least 18 years old | At least 18 years old | At least 18 years old |
|  |  | Their relative with ABI had to be at least 18 years old | Their relative with ABI had to be at least 18 years old |
|  | Service user of the collaborating organization | Their relative with brain injury had to be a user of services in the collaborating organization | Their relative with brain injury had to be a user of services in the collaborating organization |
| **Exclusion criteria** | Had speech and communication difficulties that prevented them from participating | Had speech and communication difficulties that prevented them from participating | Had speech and communication difficulties that prevented them from participating |
