# Supplementary material for: Family quality of life after brain injuries: a qualitative study on the perspectives of family members
Source: Qual Life Res. 2025 Jul 4;34(9):2701–17. doi: 10.1007/s11136-025-04011-z (PMC12432037; doi:10.1007/s11136-025-04011-z)
Supplement: Supplementary file 2 — (DOCX 244 KB) [file 11136_2025_4011_MOESM2_ESM.docx]

**Family quality of life after brain injuries: A qualitative study on the perspectives of family members**

[*Author information - Anonymized*]

# Supplemental Data File 2. Focus Groups Guide

## Before Focus Groups

Prior to beginning Focus Groups, participants were provided with the necessary information about the study’s aims, its procedures and questions about the study were solved. Also, participants were asked to provide informed consent to participate in the study. Finally, participants had to complete an *ad hoc* survey with self-reported sociodemographic and clinical information. Special attention was given to establishing a relationship in the first two sections of the group.

## Introducing Focus Groups

Moderators were introduced to the group, as well as the ground rules for the group discussion. Bellow, the rules of the group are displayed:

*Good morning! We are (…), and we will be moderating the group. As we said, we are also going to record the session as it is difficult to keep all the information, so we can take all your opinions into account. Are you okay with that?*

*We have brought you together because we would like you to discuss about such an important, and sometimes neglected, topic as the family and its wellbeing. In groups like this one, we usually find that some people talk more, and others talk less. Either way, we would like to hear from all of you, for this reason we are going to intervene in order to ask you a series of questions and try to give everyone a chance to speak and tell us what you think. So, we would like you to discuss these topics and to intervene whenever you want.*

*Also, I would like this group to be a safe space, where everyone can express freely, so I am going to ask you two things: First, please, do not comment on anything that is said within the group so that we can all express what we want comfortably. And, secondly, that we respect what everyone says. In this sense, there are no right or wrong opinions, because you are speaking from your experience and you are the experts, so we want to hear what you think.*

*Furthermore, during the group, if you have something to comment, we want you to share it with everyone, so there’s no parallel discussions.*

*Do you have any question?*

## Warm up

In this section, participants were asked to introduce themselves and their families. Also, discussion started asking about how their lives before the acquired brain injury (ABI) was and how did it change following ABI. Bellow, the questions of this section are displayed:

*Well, I would like to start the group by asking you to introduce yourselves, to describe what your family is like, how many people are in your family, who are they? (…)*

*Okay, well, now that we all know each other a little bit, I would like you to think about your family and all the people you have mentioned and tell us what, if anything, is different in your family since the brain injury occurred.*

## Main questions

In this section, participants were asked to discuss what was family quality of life (FQoL) for them, which aspects comprise it and which factors have influenced such aspects.

*Well, we have been talking about how your families have changed. Now, I would like you to tell me what comes to your mind when we talk about family wellbeing/family quality of life (…).*

*Okay, you have mentioned some aspects that are important to you, could you tell me what other aspects are part of family well-being//family quality of life.*

In this sense, specific questions were posed to clarify and provide more information about FQoL, including topics found in the previous literature and in previous groups.

*So far, we have looked at those things that seemed important to you as a part of family well-being. However, now I would like you to think about those times when things have gone really well for your families, what things have helped them to go well?*

*Well, you have told me what things helped things went well, now I would like you to tell me what things make things go badly*.

## Closing

In this section, participants were asked to summarize the topics explained within groups, as well as to provide suggestions for professionals and other families that would live their same experiences. Finally, the group was closed by thanking them for their participation.

*We are just finishing, I would like to sum up what we have been talking about, what aspects do you mention to be a part of your family well-being, and what thing could help or hinder it.*

*Okay, we have been talking about all those things, finally, I would like to know what you would say to professionals about how they can help your families//to those families in which one of their relatives is about to have a brain injury.*

*Also, we would like to thank you for your participation and ask if there is anything you would like to add, ask or comment on.*
