# Supplementary material for: Family quality of life after brain injuries: a qualitative study on the perspectives of family members
Source: Qual Life Res. 2025 Jul 4;34(9):2701–17. doi: 10.1007/s11136-025-04011-z (PMC12432037; doi:10.1007/s11136-025-04011-z)
Supplement: Supplementary file 3 — (DOCX 262 KB) [file 11136_2025_4011_MOESM3_ESM.docx]

**Family quality of life after brain injuries: A qualitative study on the perspectives of family members**

José Luis Castillo ^1, 2, *^

Alba Aza ^1, 2^

María Fernández ^1, 2^

Mari Storli Rasmussen ^3, 4, 5^

Nada Andelic ^3, 4^

Miguel Ángel Verdugo ^1, 2^

^1^ Institute for Community Inclusion (INICO), University of Salamanca, Spain

^2^ Department of Personality, Assessment, and Psychological Treatments,

University of Salamanca, Spain

^3^ Department of Physical Medicine and Rehabilitation, Oslo University Hospital,

Norway

^4^ Faculty of Medicine, Research Centre for Habilitation and Rehabilitation Models and

Services (CHARM), Institute of Health and Society, University of Oslo, Norway

^5^ Faculty of Health Sciences, Oslo Metropolitan University, Norway

^*^ Correspondence: [joseluiscastilloladeras@usal.es](mailto:joseluiscastilloladeras@usal.es)

# Supplemental Data File 1. Table 1. Consolidated criteria for reporting qualitative research (COREQ) checklist

**Table 1.** COREQ Checklist.

| **Nº of Item** | **Guide question/description** | **Page(s)** |
| --- | --- | --- |
| **Domain 1: Research team and reflexibility** | | |
| Personal Characteristics | |  |
| 1. Interviewer/facilitator | Which author/s conducted the interview or focus group? | 5-6 |
| 1. Credentials | What were the researcher’s credentials? E.g. PhD, MD | Authors information |
| 1. Occupation | What was their occupation at the time of the study? | Authors information |
| 1. Gender | Was the researcher male or female? | Authors information |
| 1. Experience and training | What experience or training did the researcher have? | Authors information |
| Relationship with participants | |  |
| 1. Relationship stablished | Was a relationship established prior to study commencement? | 5 and SM File 2 |
| 1. Participant knowledge of the interviewer | What did the participants know about the researcher? e.g. *Personal goals, reasons for doing the research* | 5 and SM File 2 |
| 1. Interviewer characteristics | What characteristics were reported about the interviewer/facilitator? e.g. *Bias, assumptions, reasons and interests in the research topic* | 5 |
| **Domain 2: Study design** | | |
| Theoretical framework | |  |
| 1. Methodological orientation and Theory | What methodological orientation was stated to underpin the study? e.g. grounded theory, discourse analysis, ethnography, phenomenology, content analysis | 5 |
| Participant selection | |  |
| 1. Sampling | How were participants selected? e.g. purposive, convenience, consecutive, snowball | 3 |
| 1. Method of approach | How were participants approached? e.g. face-to-face, telephone, mail, email | 3 |
| 1. Sample size | How many participants were in the study? | 3-4 |
| 1. Non-participation | How many people refused to participate or dropped out? Reasons? | No information |
| Setting | |  |
| 1. Setting of data collection | Where was the data collected? e.g. home, clinic, workplace | 5-6 |
| 1. Presence of non-participants | Was anyone else present besides the participants and researchers? | 5 |
| 1. Description of sample | What are the important characteristics of the sample? e.g. demographic data, date | 3-5 |
| Data collection | |  |
| 1. Interview guide | Were questions, prompts, guides provided by the authors? Was it pilot tested? | Supplemental Data File 2 |
| 1. Repeat interviews | Were repeat interviews carried out? If yes, how many? | 3-5 |
| 1. Audio/visual recording | Did the research use audio or visual recording to collect the data? | 5 |
| 1. Field notes | Were field notes made during and/or after the interview or focus group? | 5-6 |
| 1. Duration | What was the duration of the interviews or focus group? | 5 |
| 1. Data saturation | Was data saturation discussed? | 5-6 |
| 1. Transcripts returned | Were transcripts returned to participants for comment and/or correction? | 5-6 |
| **Domain 3: Analysis and findings** | | |
| Data analysis | |  |
| 1. Number of data coders | How many data coders coded the data? | 5-6 |
| 1. Description of coding tree | Did authors provide a description of the coding tree? | 6-12 |
| 1. Derivation of themes | Were themes identified in advance or derived from the data? | 5-6 |
| 1. Software | What software, if applicable, was used to manage the data? | 5-6 |
| 1. Participant checking | Did participants provide feedback on the findings? | 6 |
| Reporting | |  |
| 1. Quotations presented | Were participant quotations presented to illustrate the themes / findings? Was each quotation identified? e.g. participant number | 8-12 |
| 1. Data and findings consistent | Was there consistency between the data presented and the findings? | 6-12 |
| 1. Clarity of major themes | Were major themes clearly presented in the findings? | 6-12 |
| 1. Clarity of minor themes | Is there a description of diverse cases or discussion of minor themes? | 6-12 |

## Note: Adapted from Tong et al. (2007).

## References

Tong, A., Sainsbury, P., & Craig, J. (2007). Consolidated criteria for reporting qualitative research (COREQ): A 32-item checklist for interviews and focus groups. *International Journal for Quality in Health Care*, *19*(6), 349–357. https://doi.org/10.1093/intqhc/mzm042
